# Early interpersonal neurobiological assessment of attachment and autistic spectrum disorders

**DOI:** 10.3389/fpsyg.2014.01049

**Published:** 2014-09-23

**Authors:** Allan N. Schore

**Affiliations:** David Geffen School of Medicine, University of California Los AngelesNorthridge, CA, USA

**Keywords:** attachment, autism, interpersonal neurobiology, early intervention, right brain

## Abstract

There is now a strong if not urgent call in both the attachment and autism literatures for updated, research informed, clinically relevant interventions that can more effectively assess the mother infant dyad during early periods of brain plasticity. In this contribution I describe my work in regulation theory, an overarching interpersonal neurobiological model of the development, psychopathogenesis, and treatment of the early forming subjective self system. The theory models the psychoneurobiological mechanisms by which early rapid, spontaneous and thereby implicit emotionally laden attachment communications indelibly impact the experience-dependent maturation of the right brain, the “emotional brain.” Reciprocal right-lateralized visual-facial, auditory-prosodic, and tactile–gestural non-verbal communications lie at the psychobiological core of the emotional attachment bond between the infant and primary caregiver. These affective communications can in turn be interactively regulated by the primary caregiver, thereby expanding the infant’s developing right brain regulatory systems. Regulated and dysregulated bodily based communications can be assessed in order to determine the ongoing status of both the infant’s emotional and social development as well as the quality and efficiency of the infant–mother attachment relationship. I then apply the model to the assessment of early stages of autism. Developmental neurobiological research documents significant alterations of the early developing right brain in autistic infants and toddlers, as well profound attachment failures and intersubjective deficits in autistic infant–mother dyads. Throughout I offer implications of the theory for clinical assessment models. This work suggests that recent knowledge of the social and emotional functions of the early developing right brain may not only bridge the attachment and autism worlds, but facilitate more effective attachment and autism models of early intervention.

## INTRODUCTION

In a recent study on the developmental neurobiological basis of attachment in the journal *Neuropsychopharmacology*, researchers are boldly asserting, “Understanding the motivational basis of healthy and at-risk parenting may open new theoretical vistas and clinical opportunities and may lead to the construction of more specific interventions that can target disruptions to maternal–infant bonding at an earlier stage and in a more accurate manner” (Atzil et al., 2011, p. 11). And in a concurrent editorial in the *Journal of Child and Psychiatry* “Developmental neuroscience comes of age,” Leckman and March (2011) are articulating the well-established developmental principle that underlies the current intense interest in early assessment: “A scientific consensus is emerging that the origins of adult disease are often found among developmental and biological disruptions occurring during the early years of life” (p. 333). Researchers as well as clinicians are now eagerly looking for clinical applications of current knowledge about the critical biological and psychological aspects of early attachment experiences, both in optimal and non-optimal relational contexts.

Toward that end I have recently offered my own contributions. Over the past two decades I have articulated and expanded regulation theory in order to model how early emotionally laden attachment experiences indelibly impact and alter the early developing right brain, which for the rest of the lifespan is dominant for the non-verbal, holistic, spontaneous (unconscious) processing of emotional information and social interactions, for enabling the organism to regulate affect and cope with stresses and challenges, and thereby for emotional resilience and emotional well-being in later stages of life (Schore, 1994, 2003a,b, 2012a). Modern attachment theory (Schore and Schore, 2008, 2010) represents a neurobiological update of John Bowlby’s seminal work. The more comprehensive model, regulation theory offers an overarching interpersonal neurobiological model of the development, psychopathogenesis, and treatment of the early forming subjective self system. In a number of current publications in the clinical literature I am using my work on regulation theory as a guide for formulating early assessments of mother-infant attachment relationships (Schore, 2012a, 2013; Schore and Newton, 2012). In this work I describe my ongoing attempts to apply the theory to clinical practice in order to generate more effective evidence-based models of early assessment, intervention, and prevention.

The assessment model of regulation theory emerges from a foundation of three organizing principles. *First,* the construct of interpersonal neurobiology, that the structure and function of the mind and brain are shaped by social experiences, especially those involving emotional relationships. The fundamental core principle of the body of my work dictates that, “the self-organization of the developing brain occurs in the context of a relationship with another self, another brain” (Schore, 1996, p. 60). A number of research and clinical disciplines are now emphasizing the “relational” or “intersubjective” principle in models of human development, over the life span (Schore, 2014). Regulation theory describes the interpersonal neurobiology of attachment and the development of intersubjectivity. This clearly implies that a central focus of an early assessment is on the dynamics of the attachment *relationship*, and on the developing infant’s evolving capacity for *intersubjectivity* (Schore, 2012a; Ammaniti and Gallese, 2014).

*Second*, recent advances in our understanding of the ontogeny of brain lateralization now indicate that a critical period of growth in the right hemisphere precedes that in the left in human infancy. Two decades ago I proposed that attachment experiences specifically impact the early developing right brain (Schore, 1994). I also presented extant studies which reported that the infant’s right hemisphere begins a growth spurt in the last trimester of pregnancy and ends in the middle/end of the second year, when the left hemisphere begins its own, a period that also marks the end of the human brain growth spurt. A number of anatomical and imaging studies now document earlier maturation of the right hemisphere in prenatal and postnatal stages of human development (Gupta et al., 2005; Sun et al., 2005; Mento et al., 2010). This body of research clearly indicates that attachment transactions influence the “early life programming of hemispheric lateralization” (Stevenson et al., 2008) that underlies the dominance of the right hemisphere in the first year of life (Chiron et al., 1997).

In addition to laterality research on the developing brain, studies on the adult brain are now shifting the classical “single brain” paradigm to a “dual brain” paradigm, and exploring precisely how the two cerebral hemispheres mediate different modes of experiencing and coping with the external world. In his monumental overview of current brain asymmetry research, neuropsychiatrist Iain McGilchrist puts forth the argument that the difference between the two brain hemispheres is profound. The right and left hemispheres create coherent, utterly different, and often incompatible versions of the world with competing priorities and values, and indeed two modes of being. He concludes, when the right hemispheric mode is dominant, “we experience – the live, complex, embodied world of individual, always unique beings, forever in flux, a net of interdependencies, forming and reforming wholes, a world with which we are deeply connected” (2009, p. 31). Hecht (2014) cites even more recent lateralization research which indicates that the right hemisphere is the primary driver of the innate psychobiological need for affiliation and social connection, and thereby for emotion regulation and personal growth. The essential adaptive right brain functions of interdependence, social connection, and emotion regulation emerge out of early attachment experiences, and they need to be assessed during the critical period.

*Third*, on the basis of the principle of hierarchical brain organization I have offered a model of the ontogeny of the emotion processing limbic system, which is shaped by the mother–infant attachment relationship (Schore, 2000, 2005). It is well-established that the right cortical hemisphere, more so than the left, is particularly well reciprocally connected with limbic and subcortical regions, and that the functional maturation of cortical-limbic circuits is significantly influenced by early socio-emotional experience. In his classic volume Bowlby’s (1969) speculated about an attachment “biological control system” that regulates the limbic system and is structured as a “hierarchical mode of organization.” Following this idea in a pair of articles in the 2001 issue of the *Infant Mental Health Journal* I articulated a model of the pre- and postnatal ontogeny of the hierarchically organized limbic system, which suggests that a number of discrete limbic components come online and develop connectivity in a defined sequence in the first year (Schore, 2001a,b). The model proposes that the neuroanatomy of the emotion processing limbic system is characterized as series of hierarchically organized “control systems” or “hubs” in the right amygdala, right anterior cingulate, and right orbitofrontal cortex, (Schore, 2003a, 2012a,b, 2013).

In line with the morphogenetic principle of caudal to rostral brain development, regulation theory proscribes a maturational sequence over prenatal to postnatal stages of more complex right-lateralized control (regulatory) systems in the amygdala, then anterior cingulate, then orbitofrontal cortex. This hierarchical circuit of emotion regulation is imprinted in affective attachment transactions over the course of first year, bottom-up, that is, subcortical to cortical, amygdala at the beginning of the first year to orbital prefrontal cortical at the end of infancy. Research now demonstrates subcortical components of the limbic system (e.g., amygdala) mature earlier than the cortical components, and that their maturation is influenced by attachment experiences. It is often overlooked that the first year is a time of tremendous expansion of subcortical brain areas. Knickmeyer et al. (2008) report that the volume of the human subcortical area (including brainstem) increased by 130% in the first year of human life. Early assessments need to shift from later maturing cortical executive functions to earlier-developing subcortical functions.

Integrating the preceding three developmental organizing principles of interpersonal neurobiology, right brain lateralization, and ontogenetic progression of hierarchical regulatory systems, I offer a psychoneurobiological model that can be used to evaluate the burgeoning attachment system, as it is evolving in real developmental time. This interpersonal neurobiological assessment of social-emotional development attempts to observe and document, in any specific case, the dynamic impact of the right brain emotion transacting attachment relationship on the infant’s developing right-lateralized limbic system. Modern attachment theory also offers an heuristic model of both optimal and less than optimal early right brain development. The model suggests that optimal development in infancy reflects a growth-facilitating relational context that promotes the progression of more complex limbic control systems in the right brain that support a foundation of later emotional-wellbeing. On the other hand, less than optimal right brain development is associated with various growth-inhibiting relational contexts that generate altered subcortical-cortical patterns of limbic–autonomic connectivity, or even structural and neuropathological alterations within the regulatory hubs themselves, and thereby inefficient control functions. Interpersonal neurobiological assessments of both the infant–mother relationship and the infant’s social-emotional development should be done over the course of the first year, during the critical periods of maturation of right-lateralized amygdala, anterior cingulate, and orbitofrontal limbic regulating control systems.

But how can an individual infant’s right brain social-emotional development be compared to a psychobiological standard of optimal normative versus non-optimal “high-risk” social-emotional development? In my pair of *Infant Mental Health Journal* articles I contrasted optimal right brain development in secure attachments with non-optimal right brain development in contexts of relational trauma (abuse and/or neglect) associated with disorganized-disoriented insecure attachments. In subsequent work I utilized the perspective of regulation theory and the construct of “relational trauma” to model the role of right brain attachment trauma in the psychopathogenesis of a DSM Axis l disorder (post-traumatic stress disorder, Schore, 2003b) as well as Axis ll disorders (borderline personality disorder and antisocial personality disorder; Schore, 2002, 2003a, Meares et al., 2011).

In this paper I apply the regulation model of early assessment to another Axis l “developmental disturbance,” autistic spectrum disorders, which manifest both early appearing neurobiological disturbances and severe developmental psychopathology. In light of the longstanding gap, indeed disconnect between the attachment and autism worlds, it may seem surprising that attachment theory can make important contributions to a deeper understanding of autism. This work bears directly upon the clinical need for the early identification of what has previously been called “infantile autism.”

Indeed, echoing the call for early interventions by attachment scientists cited above, autism researchers studying “the very early autism phenotype” are now asserting, “One purpose of the earlier identification is that treatments delivered at very young ages, when the brain is most plastic, may ultimately lessen or prevent the lifelong challenges associated with autism” (Yirmiya and Ozonoff, 2007, p. 9). The fact that both fields are intensely focused on social-emotional development in the prenatal, perinatal, and postnatal stages of human infancy highlights the problem of the early differential diagnosis of these developmental disorders. In classic writings on childhood autism Rutter (1978, pp. 146–147) observed, “there is a lack of attachment behavior and a relative failure of bonding,” but also noted that “a failure of bonding is also seen in conditions other than autism” which show a different “style of social interaction.” At present there is agreement that most children who experience attachment disorders do not develop autistic spectrum disorders, and that attachment disorders are much more common than autism. That said, both fields have now embraced an epigenetic perspective, which suggests that they may share at least some common psychoneurobiological etiological factors.

In the upcoming first section of this work I describe how the first 2 years of life represents a sequence of critical periods for the relationally driven maturation of the right brain, the “emotional brain.” More and more complex rapid, spontaneous, and thereby implicit right-lateralized visual-facial, auditory-prosodic, and tactile–gestural communications lie at the psychobiological core of the emotional attachment bond between the infant and primary caregiver. These affective communications can in turn be interactively regulated by the primary caregiver, thereby expanding the infant’s developing right brain regulatory systems. Regulated and dysregulated bodily based communications can be assessed in order to determine the ongoing status of both the infant’s emotional and social development and the quality and efficiency of the infant–mother attachment relationship. In the second section I offer an overview of current research from developmental neuroscience on the early stages of autism. I also report recent studies on alterations of right brain limbic structures in developing autistic brains, as well as a growing body of research demonstrating early appearing deficits in intersubjective behavior in autistic mother-infant dyads. This work suggests that the emotion-processing limbic system and the “emotional” right brain may bridge not only attachment and autism neurobiology, but attachment and autism models of early intervention.

## CLINICAL IMPLICATIONS OF REGULATION THEORY FOR EARLY ATTACHMENT ASSESSMENTS

Neurobiologically informed models of modern attachment theory advance Bowlby’s (1969) basic tenet that attachment is biological in nature, and underscore the morphogenetic role of the attachment relationship in the structural connectivity of the right brain, the emergence of emotional regulation, and the development of the implicit subjective self. J. R. Schore and I have suggested that the current expansion of Bowlby’s seminal ideas allows for “new understandings in *clinical assessments, shaping therapeutic interventions* from relevant theory” (Schore and Schore, 2008, p. 17, authors italics). Toward that end regulation theory can be used to guide the assessment of attachment episodes of rapid, spontaneous, implicit *visual-facial, auditory-prosodic, and tactile–gestural* affective communications, in which the primary caregiver interactively regulates the infant’s internal states of peripheral and central arousal. In this section I describe current developmental neuroscience findings on the infant and maternal processing of these sensoriaffective communications. These studies are organized as a temporal sequence over the course of the first year. I also offer clinical implications of the research studies for assessments of social-emotional development. Due to space limitations I refer the reader to Schore (2012a, 2013) for specific references to the following.

With respect to *visual-facial attachment communications*, it is now established that mutual gaze is critical to early social development. The emergence of the capacity to efficiently process information from faces requires visual input to the right (and not left) hemisphere during infancy. At 2 months of age, the onset of a critical period during which synaptic connections in the developing occipital cortex are modified by visual experience, infants show right hemispheric activation when exposed to a woman’s face. Using electroencephalography (EEG) methodology, Grossmann et al. (2007) report that 4-month-old infants presented with images of a female face gazing directly ahead show enhanced gamma electrical activity over right prefrontal areas. Recent near-infrared spectroscopy (NIRS) research (perhaps the most suitable of all neuroscience techniques applicable to human infants) reveals that specifically the 5-month-old’s right hemisphere responds to images of adult female faces. By 6 months infants show a right-lateralized, left gaze bias when viewing faces, right temporal activation when looking at angry faces, and significantly greater right frontotemporal activation when viewing their own mother’s (as opposed to a stranger’s) face.

Note the developmental progression over the first year to more complex visual-affective functions. These research data indicate that the future capacity to process the essential social information expressed in face-to-face communications, a central aspect of all later intimate relationships, is dependent upon caregiver-infant eye contact and visual gazing during early critical periods. Thus, how often and in what contexts the mother and infant *spontaneously* look (and not look) directly at each other is of key importance to a clinician when evaluating both an infant’s development and the health of the dyadic relationship. When there is mutual infant–caregiver visual gazing that looks and feels natural, the clinician implicitly knows that the infant’s brain is likely developing well in this area.

Ongoing studies of prenatal, perinatal, and postnatal *auditory-prosodic attachment communications* also highlight the role of the right brain. In an EEG study of auditory pitch processing in preterm infants born at 30 gestational weeks, Mento et al. (2010) conclude, “the earlier right structural maturation in fetal epochs seems to be paralleled by a right functional development” (p. 1). A functional magnetic resonance imaging (MRI) study of 1- to 3-day-old newborns reports that music evokes right hemispheric activation in the auditory cortex. Using NIRS with 2- to 6-day-old neonates, Telkemeyer et al. (2009) observe, “responses to slow acoustic modulations are lateralized to the right hemisphere” (p. 14726). This same optical brain imaging technology reveals that prosodic processing of emotional voices in 3-month-old and 4-month-old infants activates the right temporoparietal region. Grossmann et al. (2010) report that 7-month-old infants respond to emotional voices in a voice-sensitive region of the right superior temporal sulcus, and happy prosody specifically activates the right inferior frontal cortex. These authors conclude, “The pattern of findings suggests that temporal regions specialize in processing voices very early in development and that, already in infancy, emotions differentially modulate voice processing in the right hemisphere” (p. 852). At 11-months the voice of a woman’s child-directed speech (i.e., with somewhat exaggerated prosody), elicits a right-lateralized event-related potential.

Furthermore, neuroscience now supports the principle that the caregiver’s use of infant-directed speech (“motherese”) is critical for the development of the posterior areas of the right hemisphere that process prosodic-emotional functions. Independent of culture infant-directed speech is preferred over adult-directed speech as early as a few weeks after birth. Compared to adult-directed speech, motherese, the vocal expression of emotion to infants, is higher in pitch, has a wider pitch range, and exhibits exaggerated pitch contours. In addition, it is shorter, slower, and separated by longer pauses than adult speech. Developmental neurobiological research demonstrates that maternal infant directed speech activates the right temporal area of 4- to 6-month-old infants, and that this activation is even greater in 7- to 9-month-old infants. In 11-month-old infants, the voice of a woman’s infant-directed speech (i.e., with somewhat exaggerated prosody) elicits a right-lateralized event-related potential.

Again, note the developmental progression of auditory affective functions that allow for more complex communication. The emotional quality of what infants hear in the early stages of infancy affects the development of the voice processing areas of the right hemisphere, especially the temporal voice areas in the upper banks of the right superior temporal sulcus. Clinically, these studies indicate the importance of assessing not the verbal content but the melody of the mother’s voice, and whether or not she’s using infant-directed versus adult-directed speech in her interactions with her child, especially in both arousal reducing calming-soothing and arousal amplifying playful contexts. This use of infant-directed speech is essential to the development of the infant’s right temporal areas, and the burgeoning ability of reading the emotional tone of the voice of others, is an essential element of all later social relationships.

In terms of *tactile–gestural attachment communications*, Sieratzki and Woll (1996) describe the effects of touch on the developing right hemisphere, and assert that the emotional impact of touch is more direct and immediate if an infant is held to the left side of the body (“left sided cradling”). Clinical research demonstrates the essential role of maternal “affective touch” on human infant development in the first year of life. This allows the infant and mother to create a system of “touch synchrony” in order to alter vagal tone and cortisol reactivity. The dyad thus uses “interpersonal touch” as a communication system, especially for the communication and regulation of emotional information. Other studies report high levels of tactile stimulation and mutual touch occur in breastfeeding, and an increase in EEG amplitude in right posterior cortical areas in 6-month-old infants during the intense somatosensory tactile contact of breastfeeding.

This research supports the infant’s need for affectionate touch for healthy right hemisphere development, which can be observed in an infant-caregiver assessment. Clinicians need to appraise not only the quality and amount of *spontaneous* caregiver-infant direct pupil-focused eye gazing and auditory communications but also the quality and amount of sensitive interpersonal touch the infant is receiving and expressing. Left versus right sided cradling should be assessed, since right cradling has been associated with maternal depression and maternal stress. The finding that adults who cradle on the right are more detached and less responsive to their infants than those who cradle on the left can also be used diagnostically (see Schore, 2013).

As the securely attached infant enters toddlerhood, his or her interactively regulated right brain visual-facial, auditory-prosodic, and tactile–gestural communications become holistically integrated, allowing for the emergence of a coherent right brain emotional and corporeal subjective sense of self. Confirming this model of the critical importance of right brain-to-right attachment communications in the progressive social experience-dependent lateralization of the right brain, neuroscientists now document that the right brain hemisphere is dominant in human infants, that the strong and consistent predominance for the right hemisphere emerges postnatally, and that the mother’s right hemisphere is more involved than the left in emotional processing and mothering. Studying structural connectivity asymmetry in the perinatal brain with newborn infants *at the beginning of the first year*, Michael Meaney and his colleagues conclude,

[I]n early life the right cerebral hemisphere could be better able to process … emotion (Wada and Davis, 1977; Schore, 2000). This idea appears consistent with our findings of rightward asymmetry in … limbic structures … These neural substrates function as *hubs in the right hemisphere for emotion processes and mother and child interaction* (Ratnarajah et al., 2013, p. 193, my italics)

Tronick’s recent research on infants *in the middle of the first year* reports 6-month-old infants use left sided gestures generated by the right hemisphere in order to cope with the stressful face-to-face- still face paradigm. They interpret this data as being “consistent with Schore’s (2005) hypotheses of hemispheric right-sided activation of emotions and their regulation during infant–mother interactions” (Montirosso et al., 2012, p. 826). Using NIRS Minagawa-Kawai’s et al. (2009, p. 289) study of infant mother attachment *at the end of the first year* observe, “Our results are in agreement with that of Schore (2000) who addressed the importance of the right hemisphere in the attachment system”.

Consonant with Bowlby’s deduction that the hierarchically organized attachment control system is centrally involved in the organism’s states of arousal, my work indicates that at the most fundamental level, the attachment mechanism is expressed as right brain-to-right brain interactive regulation of affective arousal, and thereby the regulation of biological synchronicity between and within organisms. Bowlby (1969, p. 156) speculated, the “upgrading of control during individual development from simple to more sophisticated is no doubt in large part a result of the growth of the central nervous system.” Earlier I described the emergence of a hierarchical sequence of interconnected limbic “control systems” or “hubs” in the right amygdala, right anterior cingulate, and right orbitofrontal cortex. These three systems interconnect with each other and with arousal regulating bioaminergic neuromodulatory dopaminergic, noradrenergic, and serotonergic nuclei in the brainstem and midbrain, as well as with neuroendocrine nuclei in the hypothalamus, the “head ganglion” of the autonomic nervous system, and therefore each inputs the stress regulating hypothalamic–pituitary–adrenocortical (HPA) axis.

Each hierarchical level of the three tiered limbic system processes and imprints a positive or negative hedonic charge on current exteroceptive information about changes in the external social environment and then integrates it with interoceptive information about concurrent alterations in internal bodily states. Although all process exteroceptive and interoceptive information, the later maturing systems in the cortex process this information in a more complex fashion than the earlier subcortical components. The output of the lowest level limbic levels is expressed as automatic innate reflexes, while higher processing produces more flexible intuitive responses that allow fine adjustment to environmental circumstances. In three dimensions, the attachment control system is hierarchically structured as an outer-later developing orbitofrontal-limbic regulated core, an inner earlier-developing cingulate-limbic regulating core, and an earliest evolving amygdala-regulated core, like nested Russian dolls.

In my most recent articulation of this model of the ontogenetic progression of hierarchical limbic–autonomic regulatory centers, I offer evidence showing that the amygdala (especially the central nucleus), the paraventricular areas of the hypothalamus that produce the stress reducing neuropeptide oxytocin and the stress intensifying neuropeptide corticotropin releasing factor, and the insula, involved in stress-responsive visceroautonomic functions, begin their maturation prenatally and are functional at birth and over the ensuing perinatal stage (Schore, 2012a). In light of the fact that the emotional state of the mother influences the fetus, assessing the emotional well-being of the mother-to-be in pregnancy is critical.

At 2–3 months the right basolateral amygdala, which densely connects with higher cortical areas, begins a critical period of growth, initiating the infant’s burgeoning intersubjective functions. From 3 to 9 months, the anterior cingulate, a cortical-limbic structure associated with responsivity to social cues comes online, giving the infant even greater capacities for intersubjectivity and for receiving non-verbal communications of “good-enough” caregiver interactive regulation. From 10 to 12 months of age the regulatory center in the orbitofrontal cortex, the attachment executive control system, begins its developmental growth period, which spans until the end of the second year (Schore, 2000). With optimal relational attachment experiences, the vertical axis that connects the right orbitofrontal cortex with subcortical areas is well developed, allowing the right orbitofrontal cortex to regulate the right amygdala.

Indeed, developmental neurobiological research reveals that the most rapid change in brain maturation occurs in the first 3–6 months of life, followed by slower change until 24 months, and relative stability after 24 months (Hermoye et al., 2006). Other studies demonstrate that amygdala function contributes to human attachment security (Lemche et al., 2006), and that the process of coping with early life stress increases the myelination of the orbitofrontal cortex that controls arousal regulation and thereby promotes emotional resilience (Katz et al., 2009). For the rest of the life span the right, and not left lateralized prefrontal regions are responsible for the most complex regulation of affect and stress (Schore, 1994; Sullivan and Gratton, 2002; Cerqueira et al., 2008; Czeh et al., 2008; Quirin et al., 2013). The right orbitofrontal cortex imprints internal representations of attachment experiences in implicit-procedural memory, thereby generating an internal working model that encodes non-conscious strategies of affect regulation. This regulatory system, the hierarchical the apex of the limbic system, is responsible for the adaptive capacities of emotional communication and regulation that is found in secure children. On the other hand functional limitations of the orbitofrontal system are seen in insecure attachment and a wide variety of psychiatric disorders (see Schore, 2003a,b, 2012a).

This ontogenetic hierarchical model suggests that right brain assessments need to be timed to crucial period transitions of the three regulatory systems (2–3, 10–12, 18–24 months), periods of complex reorganizations of the emotion processing limbic system. As the securely attached infant moves through infancy and toddlerhood, his or her interactively regulated intersubjective right brain visual-facial, auditory-prosodic, and tactile–gestural attachment communications become holistically integrated, allowing for the emergence of a right brain coherent emotional and corporeal subjective sense of self and a background state of well-being. The non-verbal implicit social-emotional capacities and deficits of both the infant’s and the mother’s right-lateralized self system are a central focus of attachment assessments. Left hemispheric verbal explicit measures cannot tap into these implicit psychoneurobiological mechanisms. In addition to ongoing clinical intersubjective evaluations, clinicians should investigate the use of current research methodologies as a potential source of developmental neuropsychological assessment tools.

## CLINICAL IMPLICATIONS OF REGULATION THEORY FOR THE EARLY ASSESSMENT OF AUTISTIC SPECTRUM DISORDERS

As previously mentioned, there is now intense interest in the problem of early intervention and assessment of not only burgeoning attachment disorders but also the earliest expression of autistic spectrum disorders, infantile autism. Autism is now viewed as a lifelong, complex neurodevelopmental disorder that severely impairs social interaction. Core symptoms include abnormal or unreciprocated interpersonal and emotional interactions, disordered social communication, and repetitive and stereotypic behaviors and restricted interests. At present it is thought that its onset most likely occurs in the latter part of the first year of life (Hazlett et al., 2005), although the field is now searching for neurobiological expressions of autism in even earlier periods of development. In a recent review Yirmaya and Charman (2010) highlight the importance of “brain development and the prodrome of autism spectrum disorders,” noting that the finding of abnormal brain growth has “attracted enormous interest.”

A large body of neurobiological studies reveals gross, widespread abnormalities in multiple areas of the autistic brain, including larger global brain volume in the first year (Courchesne et al., 2003). Autism is now characterized as a disorder of aberrant neural circuitry, and very recent research with infants at 6 months shows atypical patterns of connectivity of a number of white matter fiber tracts “not specific to any single brain region or behavioral domain” (Wolff et al., 2012, p. 596). These authors note that the earliest stages of infancy represent periods when “dramatic changes in behavior are paralleled by dramatic changes in the brain” (p. 590), and so “Extending neuroimaging downward to infants younger than 6 months would help clarify the temporal origin of diverging trajectories” (p. 597). The field is presently poised to begin more comprehensive studies of the prenatal, perinatal and early postnatal periods. Yirmaya and Charman (2010) assert, “Prenatal and perinatal environmental challenges during the course of pregnancy are likely causes of deleterious epigenetic modifications to the fetal brain with long-term developmental consequences, including risk and to autistic spectrum disorders” (p. 446).

Recall Kanner (1943) originally suggested that autistic individuals “come into the world with an innate inability to form the usually biologically provided *affective contact* with other people” (p. 250, my italics). Although the past two decades of autism research has been influenced almost exclusively by cognitive developmental psychology and cognitive neuroscience, there is currently a growing interest in altered development within autistic emotion processing “limbic circuitry” (Haznedar et al., 2000). Yirmaya and Charman (2010) observe,

The window of this abnormal brain growth coincides with the period of synaptogenesis and subsequent pruning when cortical connections are developed, refined and stabilized. *This process is interactive with the infant’s experience of the environment* … and the consequences of a developing brain whose connections are not undergoing the usual refinement on information processing … may result in … the emergence of the ASD phenotype (p. 443, my italics)

This clearly implies that updated models of autistic etiology are now shifting into the interactive relational model used in other developmental neuropsychiatric disorders, that is, the infant’s interactions with the social-emotional environment (the attachment relationship) directly influence the development of the autistic infant brain.

In their overview of the field Yirmaya and Charman (2010) refer to “an intriguing finding” that by the age of 2 years children with autism had “enlarged amygdala, particularly in the right hemisphere” (p. 443). Indeed six studies now document a “pathological enlargement of the amygdala” in autistic infants, toddlers, and young children (Sparks et al., 2002; Munson et al., 2006; Mosconi et al., 2009; Schumann et al., 2009; Kim et al., 2010; Nordahl et al., 2012). Mosconi et al. (2009) suggest “failure to orient to faces and, more specifically, the eye region of the face is inherent in multiple aspects of social impairment unique to autism (e.g., joint attention, facial emotion processing) … may be linked to amygdala abnormalities” (p. 510).

Studying children as young as 18 months Munson et al. (2006) report an association between enlarged right (and not left) amygdala volume with poorer socialization and communication development as measured on the Autism Diagnostic Interview and Vineland Scale. They speculate right amygdala enlargement may reflect “right-sided amygdala activation in response to conditioned fear” (p. 690). Commenting on this amygdala enlargement in 18-months-old toddlers Schumann et al. (2009) state, “The amygdala has long been a site of intense interest in the search for neuropathologic markers for autism, given its well-established role in the production and recognition of emotions and modulatory role in social behavior. Initial signs of autism in toddlers include unusual affective behavior, reduced social interest, and poor eye contact, which are all suggestive of aberrant amygdalar function.” (p. 942). The problem of diminished attention to faces early on “may be the first in a cascade of problems that lead to later emotional and social impairments” (p. 947). Furthermore, they conclude that the autistic toddler’s dysfunctional hyperaroused amygdala “suggests a heightened emotional, or even fearful, response when autistic individuals look at another person’s eyes, regardless of whether they are familiar or a stranger” (p. 947).

This body of research clearly implies that autistic infants and toddlers experience a chronic intense fear state in the first 2 years of infancy, and that this needs to be assessed. The finding that early developing right basolateral amygdalar enlargement, associated with amygdalar hyperreactivity and abnormal fear conditioning persists in 6-to 7-year old children (Kim et al., 2010) strongly suggest that a chronic fear-based subjectivity continues in the autistic child’s mind. My own studies on early relational trauma, disorganized attachment, and the origins of post-traumatic stress disorder implicate the right amygdala in states of fear conditioning (Baker and Kim, 2004) and “unseen fear” (Morris et al., 1999), in both hyperaroused amygdala terror states and hypoaroused amygdala dissociative, detached states of emotional withdrawal (Schore, 2001b, 2002, 2009, 2012a). This work describes not only attachment experiences of traumatic affective arousal, but the unique defenses that the infant (and mother) use to counteract traumatic hyperarousal, dissociation, “the escape when there is no escape,” “the last resort defensive strategy,” “detachment from an unbearable situation” (Schore, 2001a).

In the infant mental health and infant psychiatry literatures Guedeney and Fermanian (2001) describe the “sustained withdrawal” of the disorganized infant, manifested in frozen, absent facial expression; total avoidance of eye contact; immobile level of activity; absence of vocalization; absence of relationship to others; and the impression that the child is beyond reach. In a subsequent study Guedeney et al. (2008) observe withdrawn social behavior from as early as 2 months in “*autism*, chronic or severe pain, failure to thrive, or post-traumatic stress disorder” (p. 151, my italics). Other research reports hyporesponsivesness to social stimuli (e.g., lack of orienting to novel sounds and attention disengagement) in 6-month-old autistic infants (Baranek et al., 2013). Indeed in a case study Dawson et al. (2000) documents an infant with autism’s “checking out” after over-stimulation at 2.5 months, and presenting a “transfixed stare” during eye contact at 9 months. The right amygdala findings in autistic toddlers indicate that pathological dissociation needs to be recognized and studied by autism researchers.

Developmental neuroscience also implicates another component of limbic circuitry, the right anterior cingulate (that is strongly connected with the right amygdala) in autism. Allman et al. (2006) describe Von Economo neurons (VENs) in the anterior cingulate and fronto-insular cortex of humans that are part of the circuitry supporting social bonding. They observe that the VEN number is low at birth but increase in early development, that they are 30% more numerous in the right hemisphere than the left, and that this ratio develops in the early postnatal period. These authors state the strong and consistent predominance for the right hemisphere emerges postnatally, that this right hemisphere VEN predominance may be related to the right hemispheric specialization for the social-emotions and therefore important for normal functioning, and therefore deviations from this ratio could be dysfunctional. Thus “their emergence might be disrupted during postnatal development with dysfunctional consequences related to neuropsychiatric disorders” (p. 367). Functionally this right anterior cingulate system is involved in the fast intuitive assessment of complex social situations. Allman et al. (2006) propose, “in autism spectrum disorders the VENs fail to develop normally and that this failure could be partially responsible for the social disabilities in these disorders as a result of faulty intuition. Our theory predicts that autistic subjects will be abnormal in making intuitive decisions under conditions involving a high degree of uncertainty” (p. 372). Supporting this model they cite an MRI study of autistic individuals that reports the area of the anterior cingulate in the right hemisphere that contains the VENs is reduced in volume (Haznedar et al., 2000). I suggest that during the critical period of development of these VEN neurons some autistic infants may experience stressful levels of allostatic overload (McEwen, 2007) that induces an apoptotic destruction of these neurons of the developing anterior cingulate, an essential subsystem of right lateralized limbic circuits that evolve in the first year. This psychoneurobiological mechanism may underlie early autistic “regressive” phenomena (Yirmaya and Charman, 2010).

Von Economo neurons are also found in the human fronto-insular cortex. The right insula, a limbic structure that has extensive connections with the amygdala (Hoistad and Barbas, 2008), is involved in emotional and facial processing (Berthier et al., 1987), in integrating tonal structure with a speaker’s emotions and attitudes (Riecker et al., 2000), and in visceral and autonomic functions that mediate the generation of an image of one’s physical state (Craig, 2011). This limbic structure is activated by perceptual awareness of threat (Critchley et al., 2002), and harm avoidance (Paulus et al., 2003). It is also implicated in pain processing and serves as an alarm center, “alerting the individual to potentially distressing interoceptive stimuli, investing them with negative emotional significance” (Banzett et al., 2000, p. 2120). These functions may be severely altered in the autistic brain. Research documents that at 2 years a group of autistic toddlers exhibit sensory abnormalities, hyperesthesia or hypoesthesia to pain and touch, and hyperactivity in the somatosensory (parietal) areas of the right (and not left) hemisphere (Miyazaki et al., 2007).

Along with the right anterior cingulate, the right insula is a “hub” of a “salience network” that integrates external sensory stimuli with internal states, and thereby is implicated in subjective awareness and interoception (Craig, 2011). Autistic children show alterations in connectivity between the insula and anterior cingulate (Uddin and Menon, 2009), reflecting disturbances in affective processes and difficulty with adapting to change, restrictive and repetitive behavior, and increased sensitivity to visual, auditory, and tactile stimuli (Uddin et al., 2013). These latter authors conclude that altered connectivity between the insula and anterior cingulate underlies a dysfunction in identifying relevant internal and extrapersonal stimuli to guide behavior, and therefore “dysfunction of the system may be part of the neurobiology of autism.” Other studies of emotional face processing deficits in autism that show increased activity in salience detection in the right amygdala and insula (Monk et al., 2010). The insular dysfunction of autism thus reflects an impairment in what Craig (2010) calls “a sentient self.”

With regard to the highest level of the hierarchical emotion processing limbic circuit, Sabbagh (2004) asserts that the ability to decode others’ mental states from facial expressions is mediated by the orbitofrontal/medial temporal circuit within the right hemisphere. Offering a neurobiological model of autism he describes a “core deficit” and concludes, “The developmental roots of autism might lie in abnormal functioning of the orbitofrontal/medial temporal circuit which may, in turn, underlie the abnormal development of social-cognitive skills among individuals with autism.” Baron-Cohen, whose group first proposed the amygdala theory of autism (Baron-Cohen et al., 2000), now postulates an atypical self-representation in autism, involving dysfunctional processing in the cingulate and ventromedial (orbitofrontal) cortex (Lombardo et al., 2010).

Indeed for over two decades I have offered interdisciplinary data indicating that the right hemispheric orbitofrontal cortex and its cortical and subcortical connections support the adaptive functions of the implicit self system (see Schore, 2012a, pp. 295–296 for a large body of research confirming right hemispheric activity in generating self-awareness and self-recognition). The term “autism” is derived from the Greek word “autos,” meaning self, and since Kanner’s groundbreaking work autism has been associated with an atypical sense of self. In an extensive neurobiological overview of atypical development of self-related processes in autism spectrum disorders Lyons and Fitzgerald (2013) cite my work and others and conclude,

[T]here is substantial evidence that the main components of self-awareness including self-recognition, self-other differentiation, body awareness, theory of mind, intersubjectivity, emotion processing, language (pronoun reversal, inner speech, third person perspective), autobiographical memory and narrative self are impaired in ASD. Our review of neural substrates underlying these processes has highlighted the significance of the Right Hemisphere. (p. 753)

These authors contend that the developmental organization of the early developing self depends on relations with others, and that these early experiences are vital to right brain maturation. They conclude that “right hemisphere impairment leads to a dysfunctional self-development in autism spectrum disorders” (p, 754), and that “substantial behavioral evidence of infants who later developed autism is supporting the theory of *disrupted intersubjective behavior*” (p. 755, my italics).

In fact, a small but extremely important group of recent studies are now exploring very early deficits in “intersubjective communication” and “emotional attachment” in autistic infants. In groundbreaking clinical research using micro-analytic analysis of home videos Trevarthen and Daniel (2005) report disorganized rhythm and synchrony in the dyadic play of an 11-month-old autistic infant and her father. Their interactional analysis also reveal reduced motor capacities, as well as “abnormal withdrawal or detachment,” specifically documenting that the interaction was characterized by “long blank periods lacking in shared experience” (p. S29), clearly describing dissociative withdrawal. The authors assert that these deficits in infant intersubjectivity and mutual reciprocity that underlie “asynchronous social behavior” reflect alterations in “both brain growth and early development of skills and sociability” (p. S25).

Expanding upon this work Muratori’s et al. (2011) laboratory has offered the most productive studies of the very earliest manifestations of autism. Analyzing home videos of *spontaneous* interactions of autistic infants and their caregiver over three trimesters of the first 18 months (0–6, 6–12, and over 12 months), their video coding of interactions within a time window of 3 s includes “maintain social engagement” (operationally defined as “The infant takes up an active role within a two-way interaction in order to keep the caregiver involved. The infant interacts, vocalizes and maintains turn taking”), as well as “regulation” [“The caregiver modulates the infant’s arousal and mood; the caregiver may act to either excite (up-regulation) or calm-down (down-regulation) the infant”]. They document that autistic infants show, in the first semester, a reduction in the duration of spontaneous social behavior “such as enjoying with people and maintaining social engagement,” and “the capacity on the one hand to express and to repeat affective states, and on the other hand to be attentive and to read the affective states of the other” (p. 413). In the second semester they observe further difficulties in the development of intersubjectivity, expressed in a decreased tendency to orient to their own name, typically seen at 9 months. They conclude,

[D]ifferent intersubjective abnormalities operate during the first year to impede its development. These abnormalities reduce the typical propensity of infants and toddlers to be active in seeking interactive experiences and in sympathizing intentions and feelings of others. Side-by-side, primary caregiver-infant interactions are shaped by the *lack of infant intersubjectivity* (Muratori et al., 2011, p. 409, my italics).

In a subsequent study these researchers used more complex computational methods to analyze interaction synchrony from home movies in order to differentiate autism from intellectual disability and typical development over the first three trimesters (Saint-Georges et al., 2011). Summarizing caregiver-infant interactions in typically developing babies, they assert,

[V]ocalizations are predominant from birth … While seeking people peaks significantly at second semester … intersubjective behavior continues to grow significantly over the semesters. Thus in the second semester, a typical child is rather seeking and attending to his caregiver and little by little turns to objects … This pattern describes the typical development of shared or joint attention. (p. 10)

Describing typical caregiver behavior in these same two periods of the first year they state,

(Caregiver) vocalizations are predominant from birth. We can assume that this type of stimulation which has its roots in animal communication is the more powerful way to strengthen child attention and affective communication. Probably it happens thanks to prosodic cues specific to infant directed speech … caregiver gestural solicitations increase during the first year. (p. 10)

Note this description echoes my studies of right brain prosody and gestures.

In contrast, “autistic children show less orienting toward people in the first semester, and thereafter they exhibit a much smaller increase of seeking people behaviors” (p. 10). Importantly, orienting and being receptive toward people is not active, but passive. They conclude,

Thus, it seems that the real marker for atypical social development is the weakness in initiating a social interaction: without the increase in social initiative the ability to be receptive and responding to others also become more severe … All these results are consistent with the hypothesis of a *growing deviant development in autistic disorders*. (p. 10, my italics)

On the caregiver side, they observe that in response to a socially under-active autistic infant (lack of initiative, inability to provoke or to anticipate other’s aims, hypo-activity), the parent assumes that the infant needs to be more stimulated, and by the second trimester a parent adopts a hyper-stimulating style. Doussard-Roosevelt et al. (2003) and Trevarthen and Daniel (2005) also report parents of autistic children overstimulate their infants as a result of their inactivity. (I’d suggest this could be a relational context that increases infant dissociative withdrawal). On the basis of these data Saint-Georges et al. (2011) recommend assessment/screening in the first 6 months and parent-infant training in the second trimester.

In the most recent publication of this group Apicella et al. (2013) again report less infant motor activity in the first trimester and lower amounts of vocalizations in the second. They cite Grossmann et al.’s (2010) study on right-lateralized voice processing in 4 and 7 month infants (described in the first section of this paper), and propose, “The reduced use of vocalizations to respond could suggest that, in infants with autistic spectrum disorders, the original vocalizations do not develop into a vocal communication able to respond, engage in dialog” (p. 8). As for caregivers’ behaviors, they document a reduction in affectionate touch (less caressing or kissing) in the second trimester, suggesting that it represents a response to the infant’s atypically reduced motor and vocal development, and that this non-synchronic motor-vocal pattern may interfere with the development of reciprocity. On the other hand they also report an increase in caregiver’s stimulating gestures (gesticulating, tickling, making faces, or presenting objects to the infant), which also may be “the way parents infants with ASD adapt to their less responsive child” (p. 9).

It is interesting to note that the primary caregiver’s reduction in affectionate, loving touch may be understood as a maternal coping mechanism of defensive withdrawal in response to the intense stressor of an increasingly emotionally unresponsive autistic infant, one who is becoming more and more unable to respond to the love of the mother. In other words the intensely stressful interpersonal neurobiological context of the autistic infant–mother interaction may in some cases dyadically generate a detached “refrigerator mother.” It is frequently forgotten that a dysregulated infant may be a source of relational stress for the mother.

These studies that directly assess real-life, real-time reciprocal social interactions represent a potential paradigm shift in autism research, assessment, and treatment. Saint-Georges et al. (2011) assert that the use of engineering methods related to detecting *spontaneous* social signal processing allows “focusing on dynamic parent–infant interaction instead of single behaviors of the baby or of the parent” (p. 9). This methodology of studying interactions rather than discrete behaviors of each member of the dyad mirrors the theoretical perspective of my interpersonal neurobiological work on right brain-to-right brain intersubjective emotional communications. Trevarthen and Daniel (2005) state that the “modern diagnosis of autism” reflects the prevailing view of psychological growth as “the development of cognition,” and that to date it has ignored “the special significance of interpersonal factors” such as “infant subjectivity.” They conclude autism fundamentally represents “a failure in the integration of motives to move in purposeful ways with self awareness, and also to difficulties with intuitive awareness of other persons’ motives and states of mind. The processes that make these functions possible are not explained by cognitive neuroscience” (p. S26). For some time now both autism research and treatment have been dominated by cognitive science, cognitive neuroscience, and cognitive developmental psychology. But with a renewed interdisciplinary focus on the very early development of not left brain cognitive but right brain social-emotional survival functions and the emergence of the implicit self there is now a possibility of reforging a bridge between autism and attachment-informed treatment models, including models of early assessment and intervention.

In light of the fact that the primordial etiology of autism, attachment disorders, and indeed all early forming psychiatric disorders takes place in universally shared prenatal, perinatal, and postnatal stages of human brain development, developmental neuroscience may play a key role in this rapprochement. According to regulation theory all human infants, with whatever early genetic endowments and in both positive growth-facilitating or growth-inhibiting relational contexts, progress through the same critical periods of attachment-influenced right brain development and stages of self-organization. Both the significant degree of brain dysfunction and level of psychopathology of autistic disorders, which are essentially relational, intersubjective, social-emotional and not merely cognitive disturbances, are clearly more widespread than attachment disorders or early forming personality disorders.

The neurobiological alterations of organized insecure attachments are mainly focal alterations of cortical limbic–autonomic circuitry, while disorganized attachments show more subcortical right amygdala dysfunction. Autistic disorders involve more global alterations of various brain circuits, both subcortical and cortical. Although the right amygdala is functionally deficient in both disorganized attachment disorders and autism, early amygdala pathological enlargement and brain hypertrophy are unique to autism. It is now thought that “the processes that lead to autistic spectrum disorders probably begin during fetal development” (Abel et al., 2013, p. 391). A very recent study of RNA *in situ* hybridization of the brains of autistic children reports patches of pathological disorganization in regions mediating social and emotional functions. The authors conclude, “Our data are consistent with an early prenatal origin of autism or at least prenatal processes that may confer a predisposition to autism” (Stoner et al., 2014, p. 1216).

Based on these data I would speculate that in autism stressful alterations of the right amygdala occur *prenatally* due to untoward intrauterine influences of the social and physical environment that substantially compromise the very early development of the midbrain reticular formation – bioaminergic (dopaminergic, noradrenergic, serotonergic) nuclei that innervate the fetal amygdala. These bioamines, central to emotional functioning, have energy regulating, arousal generating, and trophic functions. For example, *in utero* early pathological mechanisms within midbrain ventral tegmental dopaminergic subnuclei could alter dopamine’s trophic functions and impair mesolimbic dopaminergic innervation of the central (and then basolateral amygdala). These systems may also be sensitive to gestational neurotoxic pesticide exposure, which has also been implicated in autism (Shelton et al., 2012).

On the other hand right amygdala alterations in disorganized attachments may result from epigenetic mechanisms associated with stressful *perinatal and postnatal* social environments. Attachment disorders reflect delayed connectivity or “immaturity” of the limbic–autonomic circuits of the right brain, whereas the “complex neurodevelopmental” autistic disorders reflect altered connectivity and “developmental derangement” of the right brain. The biological mechanisms that underlie the neuropathology of the “growing deviant development in autistic disorders” are now thought to produce an individual deviation from normative developmental processes (Jones and Klin, 2013). In autism developmental neuropathology is expressed not only in the right amygdala but in all three regulatory control systems, again suggesting a progressive metabolic failure of bioaminergic innervation, even in postnatal critical periods. Research strongly suggests that during prenatal and postnatal critical periods, aberrant DNA methylation, excessive oxidative cell damage, apoptosis, and mitochondrial dysfunction may underlie the metabolic pathology of the developing autistic brain (e.g., Melnyk et al., 2012). In any event, the earlier described progression of right brain subcortical and cortical attachment functions and structures also apply to early assessments of the ontogeny of autism.

## CONCLUSION

At the outset of this work I described a major goal of autism researchers – to create “treatments delivered at very young ages, *when the brain is most plastic,* may ultimately lessen or prevent the lifelong challenge associated with autism” (Yirmiya and Ozonoff, 2007, p. 9, my italics). The importance of early intervention in not just autism but in all developmental disorders is stressed in a number of disciplines: infant mental health, child psychology and psychiatry, pediatrics, clinical social work, and developmental neuroscience. Authors in this latter field assert, “Although the first year of life may be a period of developmental vulnerability, *it may also be a period in which therapeutic interventions would have the greatest positive affect* (Knickmeyer et al., 2008, pp. 12179–12180, my italics).”

Regulation theory highlights the potentially optimizing effects of early interventions within prenatal, perinatal, and postnatal critical periods of brain development. The theory has recently been applied to using maternal-infant interactions as a mechanism for reducing the effects of allostatic load on premature infant neurodevelopment in neonatal intensive care units (Weber et al., 2012), for differentially diagnosing attachment disorders from infantile autism in the first year (Schore, 2013; Voran, 2013), and for generating an evidence-based interpersonal neurobiological model of attachment assessments and interventions (see Schore and Newton, 2012 for applications to a clinical case, the assessment and treatment of a 7-month-old infant and his mother).

Although left hemispheric language disturbances appear later in the second and third year, the common underlying social impairment symptoms of autism spectrum disorders, abnormal or unreciprocated interpersonal and emotional interactions and disordered social communication present in the first year. In human infancy “communication” is not verbal but non-verbal communication, a specialization of the right hemisphere. The early evolving core symptomatology of infantile autism describes a developmental neuropathology and developmental psychopathology of the early developing right hemisphere, which for the rest of the lifespan is dominant for the *implicit* non-verbal, holistic processing of rapidly communicated emotional information and *spontaneous* social interactions (Decety and Lamm, 2007; Semrud-Clikeman et al., 2011; Schore, 2012a). Very recent research reveals decreased *spontaneous* attention to social stimuli in 6 -month-old infants later diagnosed with autism spectrum disorders (Chawarska et al., 2013). A developmental neuropsychological study of these implicit spontaneous functions demonstrates that “the social choices of children with autism were influenced less by emotional information presented subconsciously and suggest a subcortical contribution to the social/emotional processing deficits observed with autism” (Hall et al., 2007, p. 100). Although both the analytic left hemisphere and holistic right hemisphere are impacted by a common neuropathological process, at the core, autism represents a severe impairment of the right-lateralized implicit cortical-subcortical implicit self system that acts unconsciously, beneath levels of conscious awareness (Schore, 1994, 2003b, 2012a).

Developmental assessments of human social-emotional (as opposed to cognitive) development need to focus attention on rapid dyadic right brain-to-right brain visual, auditory, tactile (and olfactory) attachment communications that can either facilitate or inhibit the experience-dependent maturation of the infant’s developing right brain. Modern attachment theory can be used as a guide for assessing a misattuned infant/caregiver relationship by observing, experiencing and evaluating the intersubjective communication of affects and the interactive regulation of affective arousal between the infant’s and mother’s right brains (see Schore, 2012a, pp. 389–393, for schematics of regulated and dysregulated right brain attachment communications). Early chronic failures of interactive arousal regulation are expressed not only in intersubjective deficits but also in dysregulated fear-driven subjective states of consciousness in the infantile autistic brain. Regulation theory dictates that assessments of infant mental health (Schore, 2001b) in the first and second year must be relational, and need to utilize non-verbal, bodily based measures of early forming right brain intersubjective relational affectivity (implicit affect regulation), and not later forming left brain verbal language-based measures (explicit mentalization).

Early assessments of “high-risk dyads” can easily transition into clinical interventions that potentially are able to expand the mother’s implicit capacities for interactive affect regulation, the core of the attachment dynamic, thereby creating a growth-facilitating environment for infant right brain development. This work suggests that modern attachment theory can act as a catalyst for the potential cross-fertilization of the attachment and autism worlds, one that can lead to more effective clinical intervention models. In the case of infantile autism timely neurochemical interventions may also be used to prevent oxidative damage of the developing brain (e.g., Main et al., 2012) and to enhance psychological interventions (Gordon et al., 2013).

The interpersonal neurobiological perspective of regulation theory supports the clinical principle that effective early intervention during critical periods of heightened brain plasticity can facilitate the expansion of the right brain not only in infancy, but over the course of the rest of the life span. A large body of developmental psychiatric research confirms the idea that “Most mental illnesses... begin far earlier in life than was previously believed” (Insel and Fenton, 2005, p. 590). Neurobiologically informed programs of early intervention could thus bring us closer to a common goal of psychology and psychiatry, the optimization of emotional well-being and the prevention of emotional disorders.

## Conflict of Interest Statement

The author declares that the research was conducted in the absence of any commercial or financial relationships that could be construed as a potential conflict of interest.
